# Joint suppression of cardiac bSSFP cine banding and flow artifacts using twofold phase-cycling and a dual-encoder neural network

**DOI:** 10.1016/j.jocmr.2024.101123

**Published:** 2024-11-07

**Authors:** Zhuo Chen, Yiwen Gong, Haiyang Chen, Yixin Emu, Juan Gao, Zhongjie Zhou, Yiwen Shen, Xin Tang, Sha Hua, Wei Jin, Chenxi Hu

**Affiliations:** a National Engineering Research Center of Advanced Magnetic Resonance Technologies for Diagnosis and Therapy, School of Biomedical Engineering, Shanghai Jiao Tong University, Shanghai, China; b Department of Cardiovascular Medicine, Heart Failure Center, Ruijin Hospital Lu Wan Branch, Shanghai Jiao Tong University School of Medicine, Shanghai, China; c United Imaging Healthcare Co., Ltd, Shanghai, China

**Keywords:** Cardiovascular magnetic resonance, Artifact, Phase cycling, Neural networks, BSSFP, Cine, Banding, Flow, Deep learning

## Abstract

**Background:**

Cardiac balanced steady state free precession (bSSFP) cine imaging suffers from banding and flow artifacts induced by off-resonance. The work aimed to develop a twofold phase cycling sequence with a neural network-based reconstruction (2P-SSFP+Network) for a joint suppression of banding and flow artifacts in cardiac cine imaging.

**Methods:**

A dual-encoder neural network was trained on 1620 pairs of phase-cycled left ventricular (LV) cine images collected from 18 healthy subjects. Twenty healthy subjects and 25 patients were prospectively scanned using the proposed 2P-SSFP sequence. bSSFP cine of a single RF phase increment (1P-SSFP), bSSFP cine of a single radiofrequency (RF) phase increment with a network-based artifact reduction (1P-SSFP+Network), the averaging of the two phase-cycled images (2P-SSFP+Average), and the proposed method were mutually compared, in terms of artifact suppression performance in the LV, generalizability over altered scan parameters and scanners, suppression of large-area banding artifacts in the left atrium (LA), and accuracy of downstream segmentation tasks.

**Results:**

In the healthy subjects, 2P-SSFP+Network showed robust suppressions of artifacts across a range of phase combinations. Compared with 1P-SSFP and 2P-SSFP+Average, 2P-SSFP+Network improved banding artifacts (3.85 ± 0.67 and 4.50 ± 0.45 vs 5.00 ± 0.00, P < 0.01 and P = 0.02, respectively), flow artifacts (3.35 ± 0.78 and 2.10 ± 0.77 vs 4.90 ± 0.20, both P < 0.01), and overall image quality (3.25 ± 0.51 and 2.30 ± 0.60 vs 4.75 ± 0.25, both P < 0.01). 1P-SSFP+Network and 2P-SSFP+Network achieved a similar artifact suppression performance, yet the latter had fewer hallucinations (two-chamber, 4.25 ± 0.51 vs 4.85 ± 0.45, P = 0.04; four-chamber, 3.45 ± 1.21 vs 4.65 ± 0.50, P = 0.03; and left atrium (LA), 3.35 ± 1.00 vs 4.65 ± 0.45, P < 0.01). Furthermore, in the pulmonary veins and LA, 1P-SSFP+Network could not eliminate banding artifacts since they occupied a large area, whereas 2P-SSFP+Network reliably suppressed the artifacts. In the downstream automated myocardial segmentation task, 2P-SSFP+Network achieved more accurate segmentations than 1P-SSFP with different phase increments.

**Conclusions:**

2P-SSFP+Network jointly suppresses banding and flow artifacts while manifesting a good generalizability against variations of anatomy and scan parameters. It provides a feasible solution for robust suppression of the two types of artifacts in bSSFP cine imaging.

## Graphical Abstract

**Figure:**
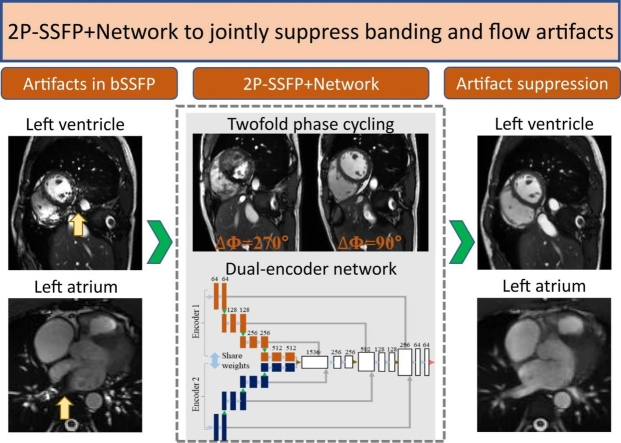

## Introduction

Cardiovascular magnetic resonance (CMR) cine imaging is essential to evaluate cardiac function, including ejection fraction, volumes, mass, and strain [1], [2], [3], [4]. For clinical cine imaging, balanced steady state free precession (bSSFP) plays a critical role due to its high signal-to-noise ratio (SNR) and contrast-to-noise ratio (CNR) [5]. However, banding and flow artifacts are common in bSSFP cine imaging and may reduce the accuracy in the assessment of left ventricular (LV) [5], [6] and left atrial (LA) [7], [8] function. Banding artifacts are caused by the off-resonance, manifested as signal voids in images [9], [10]. The flow artifacts are usually induced by time-varying out-of-slice contributions from spins flowing through the off-resonance regions, commonly appearing as signal hyperenhancement [11], [12], [13].

In standard bSSFP sequence, the transmission radiofrequency (RF) phase is incremented by 180° each TR to shift the on-resonant spins to the middle of the passband [14]. By averaging bSSFP images acquired with different RF-phase increments, one can flatten the overall bSSFP magnitude response to reduce the banding artifacts [15], [16], [17], [18]. This method is called phase-cycling bSSFP [14]. Typically, four images are required, as a linear combination of fewer phase-cycled images does not sufficiently suppress the B0-induced signal fluctuations. This results in a fourfold increase of the scan time, which is problematic for cine imaging since each bSSFP needs a single breath-hold. Furthermore, fourfold phase cycling also tends to exacerbate flow artifacts by shifting the off-resonance into flow regions [10], [19]. Partial dephasing [20] has been combined with fourfold phase cycling to reduce both banding and flow artifacts [6]; However, the proposed sequence required 21 heartbeats, and residual artifacts were still reported.

Recent evidences suggest that deep learning methods have achieved the state-of-the-art performance in suppressing various artifacts in medical images [21], [22], [23], [24], [25], [26], [27]. A neural network based on cine movies of a single RF phase increment (1P-SSFP+Network) has been proposed for suppressing banding and flow artifacts in bSSFP cine [28]. Compared with fourfold phase cycling, the 1P-SSFP+Network method reduces the scan time. However, if the artifacts occupy a large area in the original image, recovery of signals is challenging since little useful information can be gathered from the artifact-affected area. Furthermore, the method may lead to generation of anatomical features that are absent in the original images or significantly deviate from reality, a phenomenon known as “hallucinations” in the deep learning literature [29].

To address these challenges, we propose a novel method that combines a twofold phase cycling bSSFP sequence (2P-SSFP) and a neural network-based reconstruction (2P-SSFP+Network). The 2P-SSFP sequence with opposite phases provides complementary information regarding the anatomy and artifacts, resolving the issues faced by network processing based on a single input movie. The neural network-based reconstruction acts as a nonlinear combination of the source images to generate a good suppression of both artifacts. The scan needs a 12-heartbeat breath-hold, which is reasonable for most patients. To the best of our knowledge, this work is the first to combine phase cycling and neural networks to improve reliability of cine imaging.

## Materials and methods

### Twofold phase-cycled bSSFP sequence

**Fig. 1:**
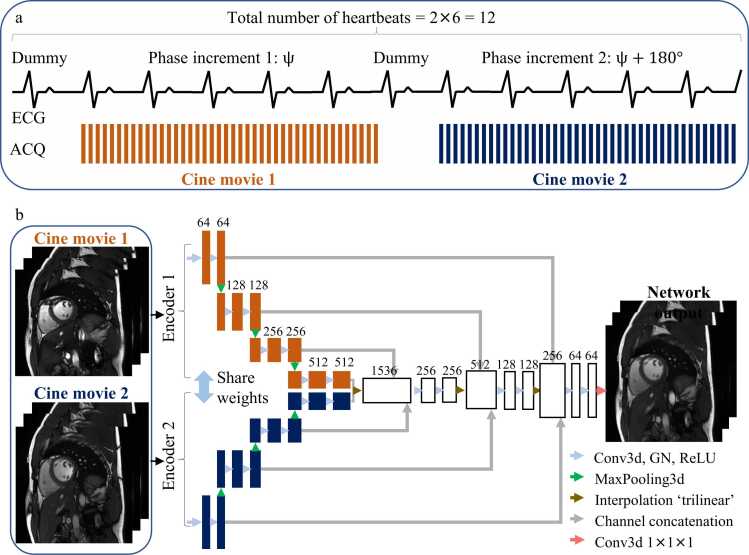
**Schematic of the proposed sequence and the neural network.** (a) An overview of the sequence design. Two cine movies with half-cycle-apart RF phase increments are sequentially acquired in a 12-heartbeat breath-hold. (b) The network adopts a modified 3-dimensional (2D+time) U-Net architecture, which involves two encoders with shared weights, each processing a single movie in the input. The number of channels is denoted on the top of the box. *RF* radiofrequency, *ECG* electrocardiogram, *ACQ* acquisition.

The twofold phase-cycled bSSFP cine sequence is illustrated in Fig. 1a. The imaging sequence performs retrospectively electrocardiogram-triggered Cartesian acquisition and sequentially acquires two cine movies with half-cycle-apart RF phase increments. In other words, if the first cine movie had a RF phase increment of ψ, the second cine movie would have a RF phase increment of ψ+ 180°. The acquisition of each cine is initialized by a dummy heartbeat to approach the steady state. Since each cine movie takes 6 heartbeats, the entire sequence lasts 12 heartbeats.

### Dual-encoder neural network

The proposed dual-encoder neural network is illustrated in Fig. 1b. The network adopts a modified 3-dimensional (2D+time) U-Net architecture [30], which involves two encoders with shared weights, each processing a single movie in the input. The fusion of feature maps at each level allows the network to integrate fully complementary information at various scales regarding the phase-cycled images. To ensure that the network produces the same result regardless of the order between the two source images, we predict the output twice using different input orders and add the results together to generate the final output. This generates a final output for any input $p_{1}$ and $p_{2}$ by

(1) $$\begin{array}{c} \mathrm{output}\left(p_{1},\;p_{2}\right)=\frac{\mathrm{network}\left(p_{1},\;p_{2}\right)+\mathrm{network}\left(p_{2},\;p_{1}\right)}{2}. \end{array}$$

which is guaranteed to be order-invariant in both training and testing. The two input images were restricted to magnitude images. Although directly operating on complex-valued images is possible, we performed all processing on magnitude images to ensure operational simplicity and compatibility with DICOM-formatted data. Additional detailed technical descriptions of the architecture can be found in Section 1 of supplemental materials.

### Study population

The study was approved by the institutional review board from each participating institution. All healthy subjects and patients provided written informed consent prior to the scan.

A total of 38 healthy subjects (mean age ± standard deviation: 23.8 ± 1.8 years, 18 females) and 25 patients (58.6 ± 15.0 years, 9 females) were scanned in two 3 T scanners located in two different institutions. The healthy subject scan was performed with a 24-channel spine coil and 12-channel torso coil, using a 3 T scanner (uMR 790, United Imaging Healthcare, Shanghai, China) located at National Engineering Research Center of Advanced Magnetic Resonance Technologies for Diagnosis and Therapy at Shanghai Jiao Tong University. The patient scan was performed with a 32-channel spine coil and 12-channel torso coil, using a clinical 3 T scanner (uMR 890, United Imaging Healthcare, Shanghai, China) located at Ruijin Hospital Lu Wan Branch. Clinical indications of these patients included ischemic heart disease (n = 1), nonischemic heart disease (n = 16), hypertension (n = 3), heart failure (n = 2), arrhythmia (n = 2), and physical examination (n = 1).

The 38 healthy subjects and 25 patients were divided into 3 datasets, namely the training dataset, the testing dataset, and the generalization dataset. Among them, the training dataset comprised of 18 healthy subjects (24.2 ± 2.2 years, 11 females), the testing dataset comprised of 20 healthy subjects (23.4 ± 1.4 years, 7 females), while the generalization dataset comprised of 10 healthy subjects (23.8 ± 1.3 years, 5 females, extracted from the testing dataset) and 25 patients. Each dataset is associated with a different imaging protocol, which is elaborated below.

### Image acquisition

#### Training dataset

For the training dataset, we performed bSSFP cine imaging in three short-axis slices: apex, mid-ventricle, and base. For each slice, we repeated the cine imaging with 12 different RF phase increments that uniformly covered the 360° range. This results in a total of 36 cine movies for each subject, which were used to train the dual-encoder network and establish the training label. Complying with the standard procedure at 3 T, we performed volumetric shimming with a shim volume slightly larger than the heart. Details about the sequence parameters are shown in Table 1.

**Table 1:** Acquisition parameters of the training and testing dataset.

	Training dataset	Testing dataset	
Sequence	bSSFP	180°-bSSFP	2P-SSFP
Scanner	uMR790	uMR790	uMR790
Imaging view, number of slices	Short-axis, 3	Short-axis, 3	Short-axis, 3
Shimming	Volumetric 3D	Automatic 2D	Automatic 2D
Number of subjects	18	20	20
FOV(RO×PE, mm)	360 × 320	360 × 320	360 × 320
Acquisition matrix (RO×PE)	224 × 199	224 × 199	224 × 199
Image matrix (RO×PE)	336 × 298	336 × 298	336 × 298
Slice thickness (mm)	8	8	8
Bandwidth (Hz/Pixel)	1000	1000	1000
TR/TE (msec)	2.86/1.31	2.86/1.31	2.98/1.37
Number of heartbeats	9	9	12
Flip angle (°)	60	60	60
Number of cardiac phases	25	25	25
Views per segment	16	24	24
RF phase increment	$\psi_{n}$*	180°	[$\psi_{n},\;\psi_{n}+180{}^{\circ}$]^+^

*bSSFP* balanced steady state free precession, *FOV* field of view, *RO* readout, *PE* phase-encoding, *TR* repetition time, *TE* echo time, *RF* radiofrequency, *2D* two-dimensional, *3D* three-dimensional.

* $\psi_{n}=360$°∙n/N, for N = 12, n = 1,2,…,12; ^+^ n = 1,2,…,6 was prescribed in 10 healthy subjects and n = 3 was fixed for other subjects; 2 P, two RF phase increments.

#### Testing dataset

For the testing dataset, we also performed bSSFP cine imaging in the 3 short-axis slices. However, instead of scanning all 12 phase-cycled cine movies, we only performed bSSFP cine with a standard 180° RF phase increment (named 180°-bSSFP hereafter) and the proposed 2P-SSFP cine sequence. For the latter, we repeated the scan 6 times, each with a pair of RF-phase increments [$\psi_{n},\;\psi_{n}+180{}^{\circ}]$, where $\psi_{n}$ for the nth scan (n=1,…,6) equals $360{}^{\circ}∙\frac{n}{12}$. To verify the robustness of the proposed method against shimming variation, we deliberately chose 2D shimming for the testing dataset, which does not need any shim volume adjustment and thus reduces the scan complexity. Details about the sequence parameters are shown in Table 1.

#### Generalization dataset

For the generalization dataset, we varied sequence parameters or scanner setups to verify the performance of the proposed method for out-of-distribution data. The dataset included both healthy subjects and patients. For the healthy subjects, we followed the same protocol as in the testing dataset after modifying the bandwidth, flip angle, imaging orientation (2-chamber and 4-chamber), and image location (left atrium) of each sequence. In the left atrium, since 180°-bSSFP often failed to provide sufficient data due to strong banding artifacts, we added a GRE cine scan to provide anatomical reference. For patients, we scanned 9 consecutive slices to construct a full short-axis stack for both the 180°-bSSFP and 2P-SSFP sequence in 15 patients. In the other 10 patients, we only scanned one short-axis slice in the mid-ventricle due to limited scan time. All other parameters of the generalization dataset were kept the same as those of the testing dataset. Details about the configuration of each subgroup are shown in Table 2.

**Table 2:** Acquisition parameters of the generalization dataset.

	Generalization dataset					
	Reduced bandwidth	Reduced flip angle	Clinical dataset	Four-chamber	Two-chamber	Left-atrium
Sequences	180°-bSSFP, 2P-SSFP, and GRE^a^					
RF phase increments	180° and [90°, 270°]					
Number of subjects	10	10	25	10	10	10
Number of slices	1	1	1 or 9^b^	1	1	4 or 5
Different parameters	Bandwidth= 500	Flip angle= 40°	Scanner: uMR890	4CH view	2CH view	LA view

*4CH* four-chamber, *2CH* two-chamber, *LA* left-atrium, *bSSFP* balanced steady state free precession, *2 P* two RF phase increments, *GRE* gradient echo.

a For the LA view, we additionally used the GRE sequence with parameters of bandwidth= 325 Hz/Pixel, TR/TE= 6.05/3.16ms, flip angle= 15°, and VPS= 10. ^b^ The number of slices was 1 for 10 patients and 9 for 15 patients, respectively.

### Network training

From the training dataset, we formed 1620 pairs of cine movies whose RF phase increments were either 90°-apart (648 pairs), 180°-apart (324 pairs), or 270°-apart (648 pairs) as the network input. We did not use only 180°-apart cine movies as the training dataset because the B0 field inhomogeneity may vary for different patients. Therefore, the combination of different RF phase increments for training may improve the generalizability of the trained model, even though during testing the network only sees cine movie pairs with opposite phases. The training labels, which were supposed to have neither banding nor flow artifacts, were generated by Short-range Phase Cycling (SPC) [28], which was the average of movies with phase increments of 120°, 150°, 180°, 210°, and 240°. The short range of phase-cycling results in a better balance between suppressing the banding artifacts and preventing the incurrence of new flow artifacts than full-range phase cycling [28]. Following the previous practice, we performed rigid image registration between the 5 cine movies during the generation of the training label to reduce potential motion that occurred between different breath-holds. Details of the training implementation are provided in Section 2 of supplemental materials.

### Network evaluation

We compared four methods, including (a) 1P-SSFP, such as 180°-, 90°-, and 270°-bSSFP with RF phase increments of 180, 90, and 270 degrees, (b) 1P-SSFP+Network, which is a network-based artifact suppression method typically using 180°-bSSFP as the input [28], (c) 2P-SSFP+Average that is an averaging of the two phase-cycled images, and (d) 2P-SSFP+Network (proposed), which is a network-based artifact suppression with two phase-cycled images of opposite RF phase increments as the input.

#### Artifact suppression performance

In the testing dataset, we firstly measured peak signal-to-noise ratio (PSNR) and the structural similarity index measure (SSIM) in the heart region on the input and output images of 2P-SSFP+Network under 6 opposite-phase combinations to show the impact of network processing. The images of short-range phase cycling served as the reference. We then invited two readers (YX and HY, with 3 and 4 years, respectively, of experience in CMR) to independently and blindly evaluate the cine movies generated from the four methods in terms of banding artifacts, flow artifacts, and overall image quality, using a 5-point Likert scale (1: non-diagnostic; 2: poor; 3: fair; 4: good; and 5: excellent). They also assessed the hallucination of the 1P-SSFP+Network and 2P-SSFP+Network methods using a 5-point scale (1: severe hallucination causing non-diagnostic quality; 2: severe hallucination with diagnostic quality; 3: moderate hallucination; 4: slight hallucination; 5: no hallucination). The input images of the networks were provided to the readers to evaluate the degree of hallucination, which is defined as either unrealistic image appearance or alteration of image content with respect to the reference image. The scores from the 2 readers were averaged to generate the final score.

#### Generalizability

We divided the generalization dataset into 6 subgroups, including reduced bandwidth (BW), reduced flip angle (FA), different clinical scanner (Clin), four-chamber (4CH), two-chamber (2CH), and left atrium (LA) imaging views. The same two readers (YX and HY) blindly evaluated the images of the three methods using the same 5-point Likert scale as above. The readers considered 5 criteria, including banding artifacts, flow artifacts, overall image quality, PV conspicuity (only for the LA), and hallucinations (only for comparison of 1P-SSFP+Network and 2P-SSFP+Network) for the generalization dataset.

#### Suppression of large-area banding artifacts

Severe off-resonance often arises in the left atrium (LA) cavity and pulmonary vein (PV) ostium, resulting in large-area banding artifacts. We compared 2P-SSFP+Network with 180°-bSSFP and 1P-SSFP+Network in the LA of 10 healthy subjects. Using GRE cine as the gold standard, we measured the average signal and coefficient of variation (COV) of each method within an ROI in the LA cavity and PV ostium. If banding artifacts are suppressed and the blood pool appears uniformly bright, the averaged signal should be high and the COV should be low.

#### Automated image segmentation

It is unclear whether artifact reduction has an impact on automated image segmentation. To investigate it, we compared the manual and automated segmentation results for 180°-bSSFP, 90°-bSSFP, 270°-bSSFP, and the proposed method in the basal, middle, and apical slices from 15 patients. One reader (CH with 9 years of CMR experience) manually traced LV endocardial and epicardial contours for each cine movie at end-diastole and end-systole using SEGMENT (Medviso, Lund, Sweden) [31]. The reader did not see any automated segmentation results to avoid potential bias. Another reader (ZC with 4 years of CMR experience) automatically traced the LV endocardial and epicardial contours without any manual interventions using SEGMENT. We then compared the manual and automated segmentation results using the Dice metric.

#### Left-ventricular ejection fraction (LVEF)

To see whether the artifact reduction affects the LVEF calculation, we evaluated LVEF in 9 short-axis slices of 15 patients for 180°-bSSFP, 1P-SSFP+Network, and 2P-SSFP+Network. For each method, the LV endocardial and epicardial contours were automatically traced without any manual interventions using SEGMENT.

### Statistical analysis

Statistical analyses were performed using IBM SPSS Statistics (version 27.0, IBM, Armonk, New York). The Wilcoxon signed-rank test was used to evaluate the qualitative score differences between different methods. The inter-reader agreement of the two readers was assessed using a two-way mixed absolute agreement intraclass correlation coefficient (ICC, <0.5 = poor; 0.5 to <0.75 = moderate; 0.75 to <0.9 = good; ≥0.9 = excellent). Paired t-test was used to evaluate the quantitative differences in the LA and PV regions between three methods. Bland-Altman plots and correlation analyses assessed the LVEF difference between different methods. P < 0.05 was considered statistically significant.

## Results

### Artifact suppression performance

**Fig. 2:**
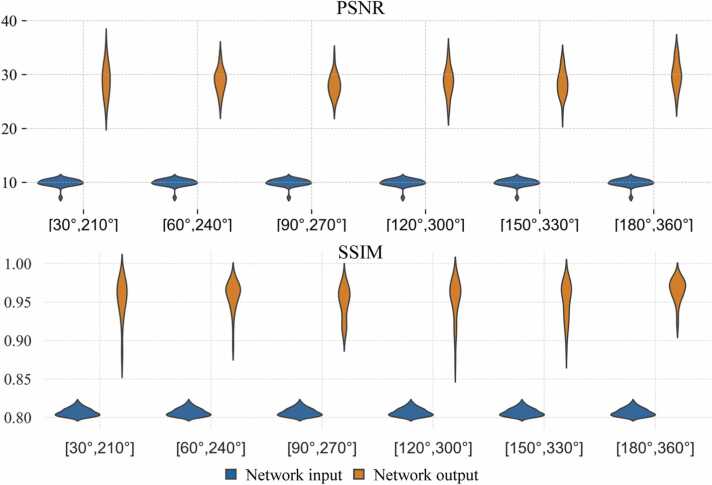
**Performance of the dual-encoder network for six different phase combinations of 2P-SSFP sequence.** The first and second rows show the PSNRs and SSIMs of the source input and output images in the heart region, respectively. The output movies had higher PSNRs and SSIMs than original cine movies, suggesting that the proposed method reduced artifacts and had a certain level of robustness against off-resonance shifts. *SPC* short-range phase cycling, *PSNR* peak signal-to-noise ratio, *SSIM* structural similarity index measure.

Fig. 2 shows PSNR and SSIM of the images in the heart before and after processing by 2P-SSFP+Network method in the testing dataset. Compared to the original cine movies, the output movies had considerably higher PSNRs and SSIMs with respect to the SPC movies. The results suggest that the proposed method reduced artifacts in six different phase combinations and had a certain level of robustness against off-resonance shifts. Although it appeared from this result that [180°, 360°] had the best performance, we chose [90°, 270°] as the default input to 2P-SSFP+Network for all subsequent processing for the testing and generalization datasets, because [90°, 270°] led to relatively mild artifacts in both source images, whereas [180°, 360°] often had strong artifacts in the 360°-bSSFP image.

**Fig. 3:**
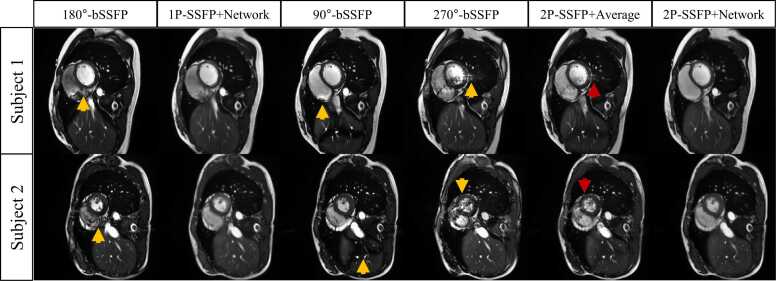
**Two examples of the four methods in the LV of two subjects.** Both 1P-SSFP+Network and 2P-SSFP+Network suppressed the banding and flow artifacts (yellow arrows), which were present in all 1P-SSFP sequences. 2P-SSFP+Average method reduced banding artifacts, yet it also generated new flow artifacts due to its simple linear combination of images (red arrows). *LV* left ventricle, *bSSFP* balanced steady state free precession, *1 P/2 P* one/two radiofrequency phase increments.

Fig. 3 shows two examples of the four compared methods in the LV of two subjects. Both 1P-SSFP+Network and 2P-SSFP+Network suppressed the banding and flow artifacts, which were present in the bSSFP images of a single RF phase increment. 2P-SSFP+Average reduced banding artifacts but also invoked new flow artifacts due to its simple linear combination.

**Fig. 4:**
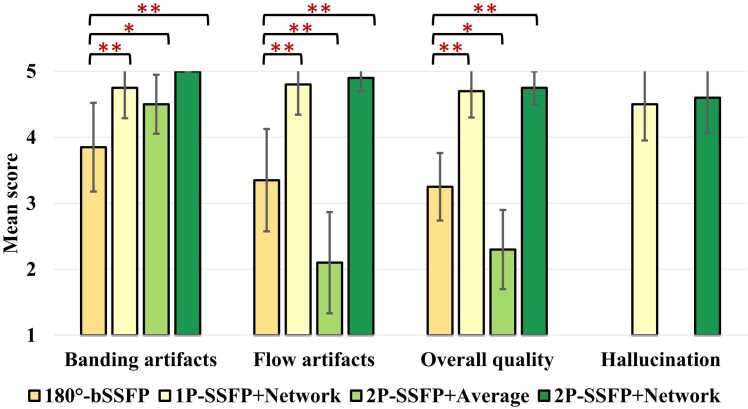
**Comparison of the qualitative scores in the testing LV short-axis data across four different methods.** Compared to 180°-bSSFP, 1P-SSFP+Network and 2P-SSFP+Network both improved banding artifacts, flow artifacts, and overall image quality. There was no significant difference between 1P-SSFP+Network and 2P-SSFP+Network. Although 2P-SSFP+Average improved banding artifacts relative to 180°-bSSFP, the former also invoked flow artifacts and impaired overall image quality. * and * * indicate P < 0.05 and P < 0.01, respectively. *bSSFP* balanced steady state free precession, *1 P/2 P* one/two radiofrequency phase increments.

Fig. 4 shows the qualitative scores across testing images for each method. Compared to 180°-bSSFP, 1P-SSFP+Network and 2P-SSFP+Network both achieved a higher score for banding artifacts (3.85 ± 0.67 vs 4.75 ± 0.46 and 5.00 ± 0.00, respectively; both P < 0.01), flow artifacts (3.35 ± 0.78 vs 4.80 ± 0.46 and 4.90 ± 0.20, respectively; both P < 0.01), and overall image quality (3.25 ± 0.51 vs 4.70 ± 0.40 and 4.75 ± 0.25, respectively; both P < 0.01). There was no significant difference between 1P-SSFP+Network and 2P-SSFP+Network methods in hallucinations (4.50 ± 0.55 vs 4.60 ± 0.54, P = 0.63). Although 2P-SSFP+Average achieved a higher score for banding artifacts (4.50 ± 0.45, P = 0.02) than bSSFP cine, the former also caused a lower score for flow artifacts (2.10 ± 0.77, P < 0.01) and overall image quality (2.30 ± 0.60, P = 0.01), suggesting that the use of a neural network for reconstruction of two phase-cycled data is necessary. ICCs of the 2 readers were 0.76 (95% CI: [0.54, 0.87]) for banding artifacts, 0.92 (95% CI: [0.86, 0.96]) for flow artifacts, 0.93 (95% CI: [0.87, 0.97]) for overall image quality, and 0.62 (95% CI: [0.43, 0.74]) for hallucinations.

### Generalizability

**Fig. 5:**
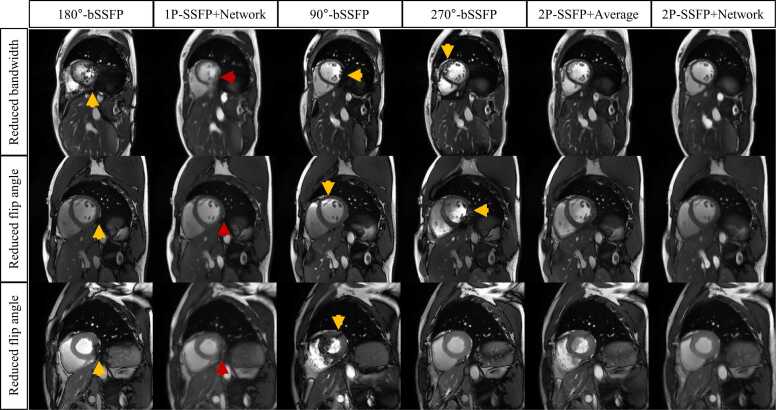
**Representative examples of four different methods in the LV cine dataset with altered sequence parameters and a different scanner.** Both 2P-SSFP+Network and 1P-SSFP+Network reduced banding artifacts and flow artifacts in the 1P-SSFP (yellow arrows). However, 2P-SSFP+Network showed a better mitigation of the banding artifacts than 1P-SSFP+Network (red arrows). While 2P-SSFP+Average also reduced the banding artifacts, flow artifacts were increased, which were not present for 2P-SSFP+Network. *LV* left ventricle, *bSSFP* balanced steady state free precession, *1 P/2 P* one/two radiofrequency phase increments.

**Fig. 6:**
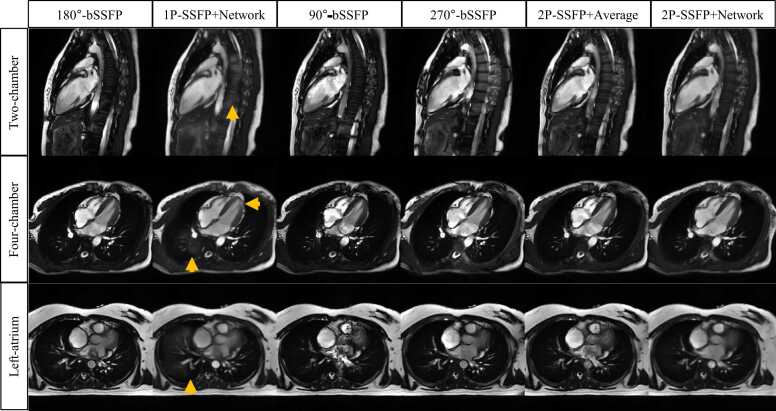
**Representative examples of four different methods with altered imaging views.** Compared to 1P-SSFP, 1P-SSFP+Network exhibited some unrealistic reconstructions in the spine, myocardium, and lung (yellow arrows), which were not found for 2P-SSFP+Network. 2P-SSFP+Average invoked flow artifacts, which were not found for 2P-SSFP+Network. *bSSFP* balanced steady state free precession, *2CH* two-chamber, *4CH* four-chamber, *LA* left atrium, *1 P/2 P* one/two radiofrequency phase increments.

Fig. 5, Fig. 6 show six representative examples of the different methods on the generalization dataset in the LV and other imaging views, respectively. In Fig. 5, both 1P-SSFP+Network and 2P-SSFP+Network reduced banding and flow artifacts relative to the bSSFP of a single RF phase increment. However, 2P-SSFP+Network showed a better mitigation of the banding artifacts than 1P-SSFP+Network. While 2P-SSFP+Average also reduced the banding artifacts, flow artifacts increased, which were not present for 2P-SSFP+Network. In Fig. 6, 1P-SSFP+Network exhibited some hallucinations in the spine, myocardium, and lung, which were not found for 2P-SSFP+Network.

**Fig. 7:**
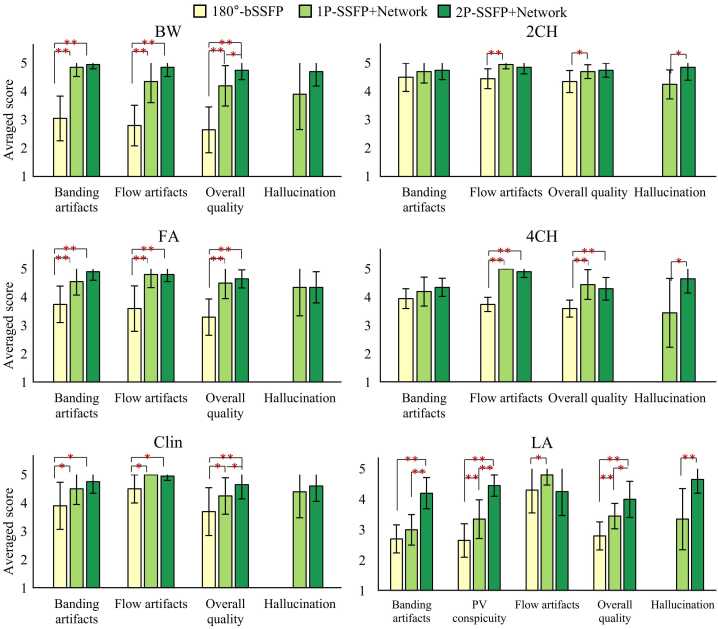
**Qualitative scores of three different methods in generalization groups.** 2P-SSFP+Network outperformed 180°-bSSFP in terms of banding artifacts (BW, FA, Clin, and LA), flow artifacts (BW, FA, Clin, and 4CH), overall image quality (BW, FA, Clin, 4CH, and LA), and PV conspicuity. 2P-SSFP+Network also outperformed 1P-SSFP+Network in terms of banding artifacts (LA), PV conspicuity, overall image quality (BW, Clin, and LA), and hallucinations (2CH, 4CH, and LA). No significant differences in flow artifacts between 2P-SSFP+Network and 1P-SSFP+Network were found for the generalization dataset. * and * * indicate P < 0.05 and P < 0.01, respectively. *bSSFP* balanced steady state free precession, *BW* bandwidth, *FA* flip angle, *Clin* clinical, *2CH* two-chamber, *4CH* four-chamber, *LA* left atrium, *PV* pulmonary vein, *1 P/2 P* one/two radiofrequency phase increments.

Fig. 7 shows the qualitative scores of the generalization dataset for the three methods. 2P-SSFP+Network outperformed 180°-bSSFP cine in terms of banding artifacts (BW, FA, Clin, and LA), flow artifacts (BW, FA, Clin, and 4CH), overall image quality (BW, FA, Clin, 4CH, and LA), and PV conspicuity. 2P-SSFP+Network outperformed 1P-SSFP+Network in terms of banding artifacts in the LA (4.20 ± 0.51 vs 3.00 ± 0.50, P < 0.01), PV conspicuity (4.45 ± 0.35 vs 3.35 ± 0.63, P < 0.01), and overall image quality in BW (4.75 ± 0.34 vs 4.20 ± 0.71, P = 0.03), Clin (4.65 ± 0.50 vs 4.25 ± 0.64, P = 0.03), and LA (4.00 ± 0.59 vs 3.45 ± 0.42, P = 0.04), suggesting a better generalizability for the proposed method. Furthermore, 2P-SSFP+Network improved the hallucination scores upon 1P-SSFP+Network in 2CH (4.85 ± 0.45 vs 4.25 ± 0.51, P = 0.04), 4CH (4.65 ± 0.50 vs 3.45 ± 1.21, P = 0.03), and LA (4.65 ± 0.45 vs 3.35 ± 1.00, P < 0.01). No significant differences in flow artifacts were found between 2P-SSFP+Network and 1P-SSFP+Network. ICCs of the 2 readers were 0.84 (95% CI: [0.71, 0.90]) for banding artifacts, 0.59 (95% CI: [−0.23,0.86]) for PV conspicuity, 0.84 (95% CI: [0.78, 0.88]) for flow artifacts, 0.84 (95% CI: [0.78, 0.88]) for overall image quality, and 0.62 (95% CI: [0.43, 0.74]) for hallucinations.

### Suppression of large-area banding artifacts

**Fig. 8:**
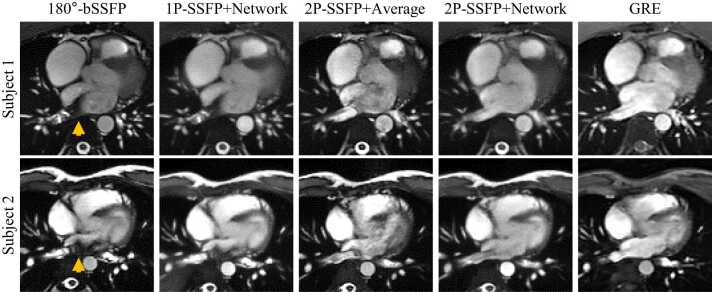
**Two representative examples of different methods in the LA.** The 1P-SSFP+Network method did not remove banding artifacts (yellow arrows) in the LA and PV regions, since it could not robustly generate correct signals for a large area. 2P-SSFP+Average method improved banding artifacts since it exploited two images of opposite phases; however, it could not robustly remove the flow artifacts and had imperfect banding suppression. 2P-SSFP+Network method well mitigated both the banding and flow artifacts, with an image quality similar to the GRE cine. *LA* left atrium, *PV* pulmonary vein, *bSSFP* balanced steady state free precession, *1 P/2 P* one/two radiofrequency phase increments, *GRE* gradient recalled echo.

Fig. 8 shows representative examples of different methods in the LA. 1P-SSFP+Network did not remove the banding artifacts in the LA and PV regions, since it cannot robustly generate correct signals for a large area. 2P-SSFP+Average improved the banding artifacts since it exploited two images of opposite phases; however, it cannot robustly remove the flow artifacts and had imperfect banding suppression. 2P-SSFP+Network well mitigated both the banding and flow artifacts, with an artifact suppression quality similar to the GRE cine.

Table 3 shows the comparison of normalized average signal and COV in the PV ostium and LA cavity for 180°-bSSFP, 1P-SSFP+Network, 2P-SSFP+Network, and GRE. The normalization was performed with respect to the average signal of the same ROI in the GRE image. For both the PV and LA, the normalized average signal of 2P-SSFP+Network was 0.51 ± 0.11 and 0.69 ± 0.15, respectively, which were significantly higher than those of 180°-bSSFP (PV: 0.22 ± 0.11, LA: 0.51 ± 0.14; both P < 0.01) and 1P-SSFP+Network (PV: 0.30 ± 0.08, P < 0.01; LA: 0.61 ± 0.17, P = 0.04). The COVs of 2P-SSFP+Network for both PV and LA were 0.21 ± 0.12 and 0.06 ± 0.02, which were significantly lower than those of 180°-bSSFP (PV: 0.52 ± 0.18, LA: 0.20 ± 0.10; both P < 0.01), significantly and trendily lower than 1P-SSFP+Network (PV: 0.30 ± 0.11, P < 0.01; LA: 0.12 ± 0.09, P = 0.06), and similar and significantly lower than GRE cine (PV: 0.16 ± 0.11, P = 0.27; LA: 0.14 ± 0.09, P < 0.01). The results suggest that 2P-SSFP+Network achieved a good suppression of the banding and flow artifacts and had uniform signals in the blood pool.

**Table 3:** Comparison of normalized average signal and coefficient of variation in the PV ostium and LA cavity for three methods.

	PV		LA	
	Normalized average signal	Coefficient of variation	Normalized average signal	Coefficient of variation
bSSFP	0.22 ± 0.11	0.52 ± 0.18	0.51 ± 0.14	0.20 ± 0.10
1P-SSFP+Network	0.30 ± 0.08	0.30 ± 0.11	0.61 ± 0.17	0.12 ± 0.09
2P-SSFP+Network	0.51 ± 0.11^a,b^	0.21 ± 0.12^a,b^	0.69 ± 0.15^a,b^	0.06 ± 0.02^a^
GRE		0.16 ± 0.11^a,b^		0.14 ± 0.09

^a^ and ^b^ denote significant difference compared to 180°-bSSFP and 1P-SSFP+Network, respectively; *PV* pulmonary vein, *LA* left atrium, *bSSFP* balanced steady state free precession, *1 P/2 P* one/two radiofrequency phase increments, *GRE* gradient recalled echo.

### Automated image segmentation

**Fig. 9:**
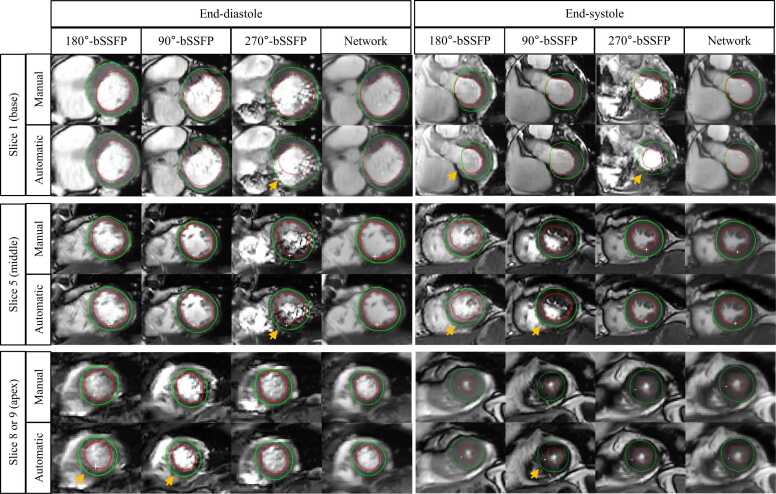
**Six representative slices of LV segmentation results for 4 methods at the end-diastole and end-systole phases.** For each slice, the first and second rows show the manual and automated segmentation results, respectively. The red and green contours denote the LV endocardial and epicardial borders, respectively. Yellow arrows denote the inaccurate automated segmentation. “Network” represents the 2P-SSFP+Network method. *bSSFP* balanced steady state free precession, *2 P* two radiofrequency phase increments.

Fig. 9 shows six representative examples of LV segmentation results for 4 methods at the end-diastole and end-systole phases. It can be seen that our method suffers from fewer artifacts than bSSFP cines with a single RF phase increment, thereby having the potential to improve automated segmentation of the myocardium. Table 4 shows the Dice metric for each method at the end-diastole and systole phases. At end-diastole, our proposed method achieved a higher Dice value compared to 90°-bSSFP (LV myocardium: 0.784 ± 0.086 vs 0.752 ± 0.113, P = 0.03) and 270°-bSSFP (LV cavity: 0.927 ± 0.034 vs 0.903 ± 0.071, P = 0.03; LV myocardium: 0.784 ± 0.086 vs 0.741 ± 0.103, P = 0.02). At end-systole, the method’s Dice value was significantly higher relative to 90°-bSSFP (LV cavity: 0.848 ± 0.092 vs 0.808 ± 0.159, P = 0.03; LV myocardium: 0.823 ± 0.061 vs 0.773 ± 0.113, P = 0.006) and 270°-bSSFP (LV myocardium: 0.823 ± 0.061 vs 0.775 ± 0.086, P = 0.001). Compared to 180°-bSSFP, the clinical standard choice, our method also led to trended higher Dice values in all circumstances (P = 0.06, 0.11, 0.07, and 0.11, from left to right), suggesting an overall improvement of segmentation quality by our method.

**Table 4:** Dice values for each method at the end-diastole and systole phases.

	End-diastole		End-systole	
	LV cavity	LV myocardium	LV cavity	LV myocardium
180°-bSSFP	0.908 ± 0.063	0.755 ± 0.111	0.794 ± 0.202	0.793 ± 0.124
90°-bSSFP	0.916 ± 0.098	0.752 ± 0.113*	0.808 ± 0.159*	0.773 ± 0.113^+^
270°-bSSFP	0.903 ± 0.071*	0.741 ± 0.103*	0.811 ± 0.115	0.775 ± 0.086^+^
2P-SSFP+Network	**0.927 ± 0.034**	**0.784 ± 0.086**	**0.848 ± 0.092**	**0.823 ± 0.061**

*bSSFP* balanced state free precession, *2P* two radiofrequency phase increments.

* and ^+^ denote P < 0.05 and P < 0.01 compared to the 2P-SSFP+Network method, respectively.

### LVEF

LVEF of the artifact-suppressed movies of 2P-SSFP+Network significantly correlated with that of the 180°-bSSFP ($R^{2}$=0.87) and 1P-SSFP+Network ($R^{2}$=0.91). Bland-Altman analyses show that 2P-SSFP+Network reduced LVEF relative to 180°-bSSFP (mean bias=−0.33%) and 1P-SSFP+Network (mean bias=−1.33%) (cf. Fig. S1).

## Discussion

In this work, we propose a combination of the 2P-SSFP sequence with a network-based reconstruction to jointly suppress banding and flow artifacts in bSSFP cine imaging. 2P-SSFP+Network reduced banding and flow artifacts relative to 1P-SSFP and 2P-SSFP+Average. In addition, the proposed method also manifested a better generalizability against variations of sequence parameters and imaging views compared to 1P-SSFP+Network. For large-area banding artifacts in the PV ostium and LA cavity, the method well suppressed these artifacts, whereas 1P-SSFP+Network did not. Finally, the method generated more accurate automated segmentation than the 1P-SSFP methods, suggesting its potential to improve accuracy of LV function assessment.

A reliable suppression of banding and flow artifacts is desirable due to the common presence of B0 inhomogeneity in the chest region. Traditional techniques, such as frequency scout [32], [33], can reduce banding and flow artifacts to a certain extent. However, as a 2D technique, frequency scout can only tell the best frequency near a single 2D slice rather than in the whole heart. In fact, the best frequency for a certain slice may be a sub-optimal frequency for other slices. Fig. S2 shows the results for regular bSSFP with an inter-TR RF phase increment of 180°, 90°, and 270°, representing a rough case of frequency scout, and the results of our proposed method (2P-SSFP+Network) in a single patient. Although each scout image always has some well-shimmed regions, these regions are not the same, requiring multiple frequency-scouts to be performed to fulfill whole-heart shimming. This approach, however, would considerably increase the workload and complicate the scan, and is rarely used in a busy clinical setting. On the other hand, our approach did not cause any substantial artifacts in any of these slices even without volumetric shimming (cf. Fig. S2). This is because our approach directly combines two cines of opposite phase increments, which already incorporate information involved in the manual frequency-scouting process. Therefore, our approach is more efficient and has the potential to even retire these extra procedures such as manual volumetric shimming and frequency-scouting, which are typically required for 3 T cardiac scans. A better suppression of the artifacts can improve the accuracy of automated segmentation as supported by our results. The improved automated segmentation can then generate more accurate LVEF, which is important for clinical evaluation of LV function.

Our approach may also find applications in the LA function assessment. The assessment of contractile function of PVs and LA can serve as important imaging markers in many diseases and procedures affecting LA, such as atrial fibrillation [34], [35], [36], [37] and the RF ablation therapy [38], [39]. However, it is known that off-resonance differs between PVs and the LA cavity by as much as 163 ± 73 Hz at 3 T [8], which often induces large-area signal voids in the bSSFP LA cine. Although GRE cine can be a reasonable substitute in this case, it has a lower SNR and myocardium-to-blood contrast, a higher susceptibility to flow artifacts, and is less used in clinical practice. Our experiments in the LA show that the proposed method performed well in this region, generating uniform signals and substantially fewer artifacts and dropouts. To our knowledge, this is the first work reporting the use of bSSFP cine in the LA and PVs without substantial banding and flow artifacts. Although previous studies have reported the reduction of banding in the LA by using 4P-SSFP [8], flow artifacts were increased causing a suboptimal image quality.

Note that when the magnetic field is further increased (e.g. at 5 T or 7 T) [40], or when the patients had cardiac implantable electronic devices (e.g. with a cardioverter-defibrillator) [41], [42], the B0 inhomogeneity would be further exacerbated. Regular bSSFP cine in these cases is likely infeasible, and GRE cine is the only solution offered so far. Our approach has the potential to improve the image quality of bSSFP cine in these extreme cases, although more experiments are needed to verify this theoretical value.

We have proposed a 1P-SSFP+Network method to jointly reduce banding and flow artifacts for cine imaging [28]. The method has advantages in that it does not increase scan time at all and can be used retrospectively to reduce the artifacts. However, while the method performed overall well in the short-axis view, where majority of training data is used, the method may result in hallucinations in other views, such as the long-axis views and transversal views, due to the change of anatomy and insufficient training data. Moreover, the method is not able to suppress artifacts in the LA, due to the presence of large banding. 2P-SSFP+Network has a better robustness against these cofounders, due to the inclusion of two images of opposite phase increments in its input. Similar techniques based on neural-network processing of multi-contrast images to reduce artifacts have also been reported in other applications, such as motion correction [43], [44], whose findings are well aligned with ours.

A disadvantage for 2P-SSFP+Network is the increase in scan time. Although the 12-heartbeat breath-hold is a reasonable time used also by e.g. T1 mapping [45] and segmented late gadolinium enhancement [46], it may still increase the likelihood of motion artifacts. To overcome this challenge, a wealth of acceleration methods can be used, including those accelerating along the temporal dimension, such as UNaliasing by Fourier-encoding the OverLaps in the temporal Dimension (UNFOLD) [47] and k-t SENSE [48], along the phase encoding direction, such as compressed sensing (CS) [49] and deep learning [50], [51], and even along the phase-cycling dimension by using an interleaved acquisition, similar to that in [6], [8]. Combination of 2P-SSFP+Network with these acceleration techniques is promising to reduce the scan time or increase the resolution.

## **Limitations**

Our technique has limitations. Firstly, as we discussed previously, the scan time is relatively long for a breath-hold and needs to be further reduced for routine employment in a clinical setting. Secondly, to ensure a 12-heartbeat scan time, we used a lower temporal resolution of 71.5 ms in the prospective cine imaging, which is insufficient for the evaluation of wall motion in systole. Again, improvement of the temporal resolution needs additional accelerations to be performed. Thirdly, we found slight blurring from the 2P-SSFP+Network output movies relative to the input. Potential causes of blurring include the lack of capability for the employed architecture to preserve fine-grained features [52] and the intrinsic blur of the training labels due to a combination of images acquired at different breath-holds. A quantitative evaluation of the degree of blurring was performed, for which the experimental details and results can be found in Section 3 of supplemental materials. Overall, we found that 2P-SSFP+Network resulted in less blurring compared with 1P-SSFP+Network, but slightly more blurring than 180°-bSSFP.

Our study also has limitations. Firstly, although significant differences were found, the number of subjects was still relatively low. More patients should be included in future studies to fully investigate the performance of the technique across different diseases and sequence parameter variations. Secondly, only cines from a single vendor at two centers were evaluated. To comprehensively analyze the generalizability of the method, cine data of subjects from multiple vendors at multiple clinical centers should be included. Thirdly, we only scanned GRE cine in the LA slice. For the most rigorous study design, GRE cine should be performed in all imaging slices to provide an anatomical reference free of banding artifacts. Fourthly, we only extensively tested a single architecture for the artifact-suppressing network, which is a 3D convolutional network. We investigated the change of the 3D architecture to an identical 2D architecture, where we found flickering artifacts in the output cine movie, suggesting that the 3D architecture has a better temporal consistency compared with the 2D architecture. As a proof of concept, investigation of more advanced architectures with potentially a better performance in preserving the fine-grained image features is left to the future.

## Conclusions

In conclusion, we propose a 2P-SSFP sequence with a network-based reconstruction method for the joint suppression of banding and flow artifacts in bSSFP cine. The method well suppresses the two types of artifacts for different sequence parameters, imaging views, and even in the LA, with a 12-heartbeat breath-hold and no manual shimming. The method provides a feasible solution for robust suppression of the banding and flow artifacts in bSSFP cine imaging.

## Ethics approval and consent to participate

The study of the healthy subjects was approved by Shanghai Jiao Tong University Ethics Review Board, and all participants provided informed written consent. The patient study was approved by Ruijin Hospital Ethics Committee of Shanghai Jiao Tong University School of Medicine, and all patients provided informed written consent.

## Funding

This work was partially supported by the 10.13039/501100001809National Natural Science Foundation of China (No. 62001288) and the Shanghai Science and Technology Commission (No. 22TS1400200).

## CRediT authorship contribution statement

**Zhuo Chen:** Writing – review & editing, Writing – original draft, Validation, Methodology, Formal analysis, Data curation, Conceptualization. **Chenxi Hu:** Writing – review & editing, Writing – original draft, Validation, Supervision, Methodology, Funding acquisition, Formal analysis, Conceptualization. **Haiyang Chen:** Methodology, Formal analysis. **Yiwen Gong:** Writing – review & editing, Methodology, Formal analysis, Data curation. **Yixin Emu:** Formal analysis, Data curation. **Zhongjie Zhou:** Data curation. **Juan Gao:** Data curation. **Xin Tang:** Data curation. **Yiwen Shen:** Data curation. **Wei Jin:** Writing – review & editing, Supervision, Funding acquisition. **Sha Hua:** Writing – review & editing, Supervision, Funding acquisition.

## Declaration of Competing Interest

The authors declare the following financial interests/personal relationships which may be considered as potential competing interests: Xin Tang reports a relationship with United Imaging Healthcare Co., Ltd. that includes: employment. If there are other authors, they declare that they have no known competing financial interests or personal relationships that could have appeared to influence the work reported in this paper.

## Data Availability

The datasets used during the current study are available upon reasonable request. The codes and trained models are openly available on Github (https://github.com/SJTU-CMRLab/2PC_Network).

## Footnotes

Appendix A Supplementary data associated with this article can be found in the online version at doi:10.1016/j.jocmr.2024.101123.
