# Supplementary material for: Joint suppression of cardiac bSSFP cine banding and flow artifacts using twofold phase-cycling and a dual-encoder neural network
Source: J Cardiovasc Magn Reson. 2024 Nov 7;26(2):101123. doi: 10.1016/j.jocmr.2024.101123 (PMC11663763; doi:10.1016/j.jocmr.2024.101123)
Supplement: Supplementary file 1 — Supplementary material [file mmc1.docx]

**Supplemental materials**

**Section 1: Architecture of the dual-encoder neural network**

The dual-encoder neural network (Fig. 1b) uses a U-Net as its backbone but has two weight-shared encoders. The two encoders' different colors represent that each encoder processes each cine movie. Each encoder consists of four levels, each with two consecutive blocks, and each block includes a 3×3×3 three-dimensional (3D) convolution layer (stride=1, padding=1), followed by a group normalization layer (the number of groups=32) [1], and a ReLU activation function. The downsampling layer adopts a 3D max-pooling with a kernel size and stride of 1×2×2 (temporal×height×width). Every step in the decoder consists of an upsampling of the feature map, a concatenation with the corresponding feature map from the encoder stage, and two consecutive blocks, the same as the block in the encoder. The upsampling layer in the decoder uses the trilinear interpolation with a scale factor of 1×2×2 (temporal×height×width). The output layer consisted of a 1×1×1 3D convolution layer. The number of channels is denoted on the top of the box in Fig. 1b.

**Section 2: Training details**

All cine images were interpolated, cropped, and normalized to enforce a uniform resolution of 1.5mm×1.5mm, image size of 192×192, and dynamic range of [0, 1]. Data augmentation was performed with two-dimensional image rotations, image translations, flipping, and elastic transforms [2]. The loss function for the network was a linear combination of mean squared error and the perceptual loss [3]. The coefficients of this linear combination were manually optimized. Training was performed with ADAM with a learning rate of 0.0001. The batch size was 1. The network was implemented with PyTorch (version 1.11.0). Training was performed over 400 epochs on a server equipped with a GPU (Tesla A100, NVIDIA, California, USA).

**Section 3: Image sharpness measurement**

To explore the blurring of the proposed network, we compared the image sharpness of different methods, including 90°-bSSFP, 1P-SSFP+Network, and the proposed 2P-SSFP+Network. We measured the image sharpness based on a previously published method [4], which measures the maximal gradient of the cross-section intensity profile across the endocardial border of the septum, at the same location for each method. We evaluated the middle slices of short-axis cine movies at end-diastole and end-systole phases in the testing dataset. Supplemental Table S1 shows the image sharpness results of the three methods. Compared to 90°-bSSFP, the 2P-SSFP+Network and 1P-SSFP+Network methods had lower image sharpness at end-diastole (0.065±0.073 vs 0.050±0.058 and 0.039±0.056, P=0.029 and P=0.015, respectively) and end-systole phases (0.052±0.031 vs 0.042±0.023 and 0.032±0.021, P=0.067 and P=0.020, respectively). Compared to 1P-SSFP+Network, the 2P-SSFP+Network method achieved higher image sharpness at the end-systole phase (P=0.045). To summarize, the proposed method introduced slightly more blurring compared to source 90°-bSSFP images. However, as a comparative study, it reduced blurring compared to 1P-SSFP+Network, which was a previous state-of-the-art artifact suppression method for bSSFP cine images [5].

**Supplemental Table S1.** The image sharpness comparison for three methods

|  | End-diastole | End-systole |
| --- | --- | --- |
| 90°-bSSFP | 0.065±0.073 | 0.052±0.031 |
| 1P-SSFP+Network | 0.039±0.056^*^ | 0.032±0.021^*+^ |
| 2P-SSFP+Network | 0.050±0.058^*^ | 0.042±0.023 |

^*^ and ^+^ denote P<0.05 compared to 90°-bSSFP and 2P-SSFP+Network, respectively.


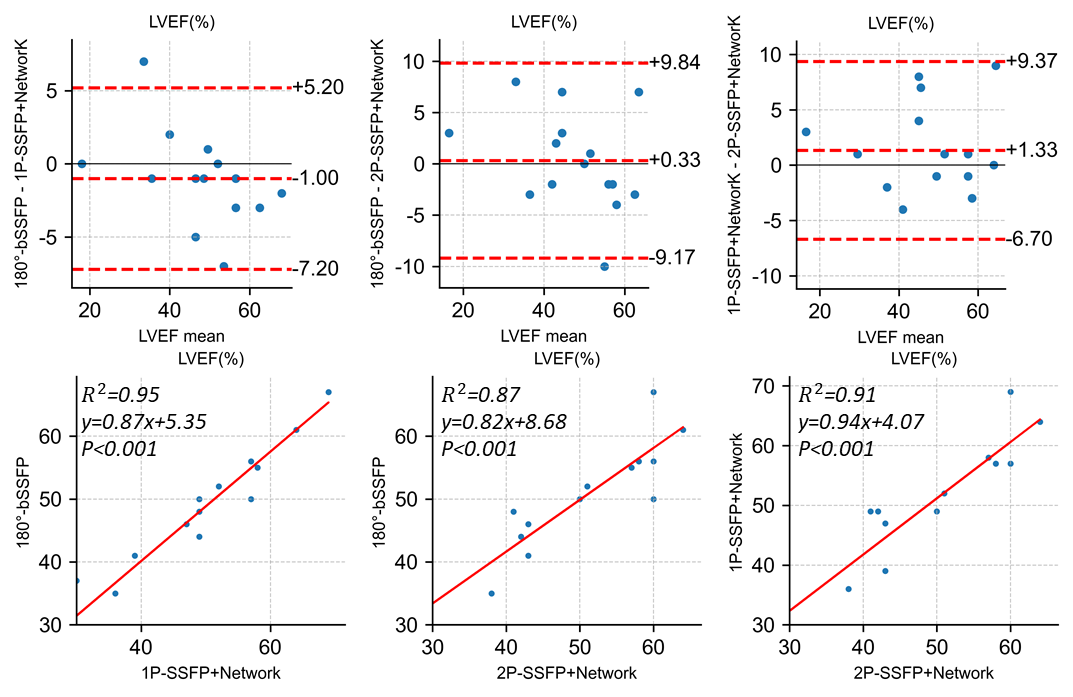


**Supplemental Fig. S1. The Bland-Altman and correlation analyses between three methods on LVEF of 15 patients.** In the Bland-Altman analysis, the red dotted lines represent the mean difference and limits of agreement (±1.96 SDs).

**
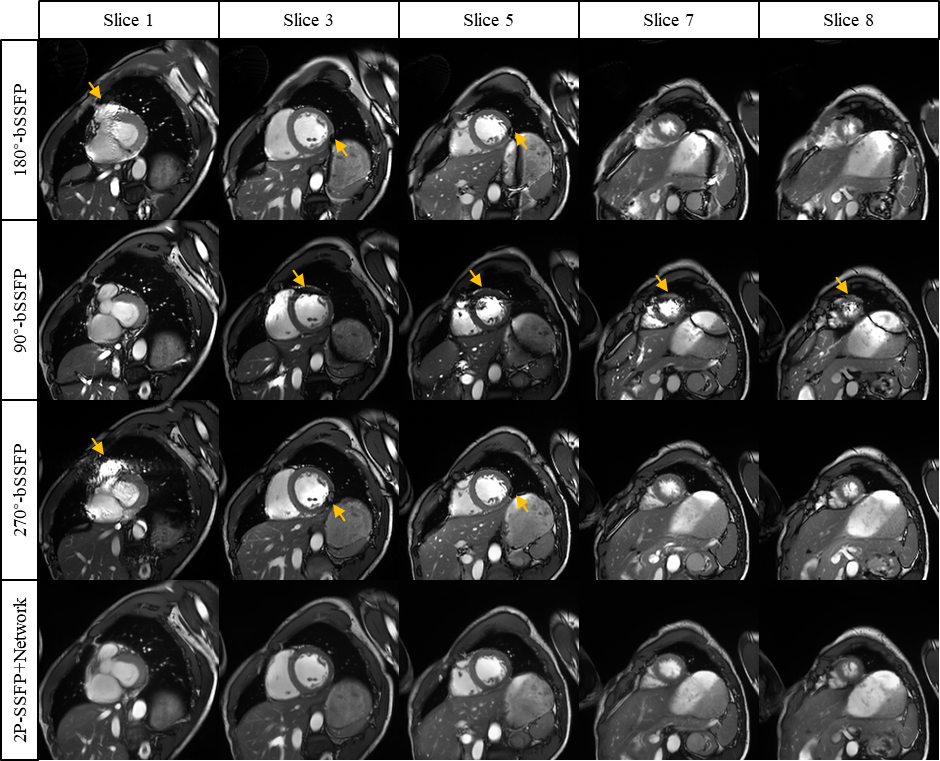
**

**Supplemental Fig. S2. Multi-slice bSSFP images with different RF phase increments and the proposed method of a single patient.** Yellow arrows denote artifacts.

**References**

1. Wu Y, He K. Group Normalization. 2018. p. 3–19. Available from: https://openaccess.thecvf.com/content_ECCV_2018/html/Yuxin_Wu_Group_Normalization_ECCV_2018_paper.html

2. Simard P, Steinkraus D, Platt J. Best practices for convolutional neural networks applied to visual document analysis. Icdar. 2003;

3. Dosovitskiy A, Brox T. Generating Images with Perceptual Similarity Metrics based on Deep Networks. Adv Neural Inf Process Syst [Internet]. Curran Associates, Inc.; 2016. Available from: https://proceedings.neurips.cc/paper/2016/hash/371bce7dc83817b7893bcdeed13799b5-Abstract.html

4. Yoon S, Nakamori S, Amyar A, Assana S, Cirillo J, Morales MA, et al. Accelerated Cardiac MRI Cine with Use of Resolution Enhancement Generative Adversarial Inline Neural Network. Radiology. 2023;307:e222878.

5. Chen Z, Hua S, Gao J, Chen Y, Gong Y, Shen Y, et al. A dual-stage partially interpretable neural network for joint suppression of bSSFP banding and flow artifacts in non-phase-cycled cine imaging. J Cardiovasc Magn Reson. 2023;25:68.
